# Risk-Benefit Comparison Between Endoloop and Endostapler Devices for the Closure of Appendiceal Stumps in Laparoscopic Appendectomy

**DOI:** 10.7759/cureus.56700

**Published:** 2024-03-22

**Authors:** Carlos Alberto Leal Hidalgo, Kevin Joseph Fuentes Calvo, Sara Fernanda Arechavala Lopez, David Jimenez Collado, José Manuel Correa Rovelo, Amado de Jesús Athie Athie

**Affiliations:** 1 General Surgery, Facultad Mexicana de Medicina, Universidad La Salle México, Mexico City, MEX; 2 Surgery, Hospital Medica Sur, Mexico City, MEX; 3 Medicine, Universidad Autónoma Metropolitana, Mexico City, MEX; 4 Ophthalmology, Institute of Ophthalmology "Conde de Valenciana", Mexico City, MEX

**Keywords:** laparoscopic appendectomy, endostapler, endoloop, appendiceal stump, appendicitis

## Abstract

Introduction

Acute appendicitis is a common cause of acute abdomen and the most frequent surgical emergency in the world. Since the nineteenth century, surgical resolution has been the most accepted treatment worldwide, and laparoscopic appendectomy is currently preferred as the treatment of choice because it has several benefits. The closure of the appendiceal stump is the most crucial step during appendectomy since its inadequate management can cause post-surgical complications. Throughout recent years, several methods have been proposed to perform this closure. This study was performed to compare the post-surgical outcomes of the use of endoloop and endostapler devices.

Methods

This is a retrospective study of 290 patients aged 18 to 83 who underwent laparoscopic appendectomy between 2016 and 2020. Demographic data, clinical history, tomographic findings, and laboratory data were collected, as well as appendicular base management technique, severity degree of appendicitis at hospital admission, postoperative complications at 30 days, hospital readmission, and in-hospital stay. Statistical tests and binary logistic regression analyses were used to identify risk factors, with a significance level of p<0.05.

Results

Demographic data and clinical history did not show statistically significant differences. The presence of a pre-surgical abscess with tomography was 1.58 times higher in the endostapler group. Post-surgical results showed that the use of endostapler devices represented a 2.7 times higher risk of post-surgical abscess. The endostapler group was also found to have 1.87 times the risk of post-surgical sepsis.

Conclusion

Our study shows that the use of an endoloop reduces the risk of postoperative abscess by 16.5% and protects against the development of post-surgical sepsis by 30%.

## Introduction

Acute appendicitis is one of the leading causes of abdominal pain, the most frequent surgical emergency in the world, and poses a significant burden to public health worldwide. The lifetime incidence of appendicitis is 8.6% for females and 6.7% for male patients; however, the risk of being subjected to an appendectomy is lower in men than in women. Peak incidence occurs between 10 and 30 years of age [1].

Since the nineteenth century, surgical resolution has been the most widely accepted treatment worldwide, with more than 300,000 appendectomies performed annually in the United States [2]. Laparoscopic appendectomy is currently the treatment of choice since it has several benefits, mainly based on its being a minimally invasive procedure, resulting in shorter hospital stays, a lower incidence of wound infection, and lower morbidity and mortality [3]. However, it is not exempt from complications like intra-abdominal abscess and ileus [4].

Closure of the appendicular stump is the most crucial step during appendectomy since its inadequate management can lead to postoperative complications, including surgical reintervention. Over time, various methods have been developed for the closure of the appendicular residual limb, and some factors that influence the selection of a closure method are the ease of application, the severity of the swelling of the residual limb, and the cost and availability of the method [5]. Some of the methods for closure of the appendicular residual limb are endostapler devices, endoloop, hem-o-locks, metal clips, bipolar coagulation, polymeric clips, and intracorporeal sutures [6-7].

All these alternatives have advantages and disadvantages during the different clinical phases of acute appendicitis, and none of them has been evaluated by prospective randomized (and experimental) studies. This study was performed to compare post-surgical outcomes between the use of endoloop and endostapler devices for the closure of the appendiceal stump in laparoscopic appendectomy in a surgical reference center in Mexico City.

## Materials and methods

**Figure 1:**
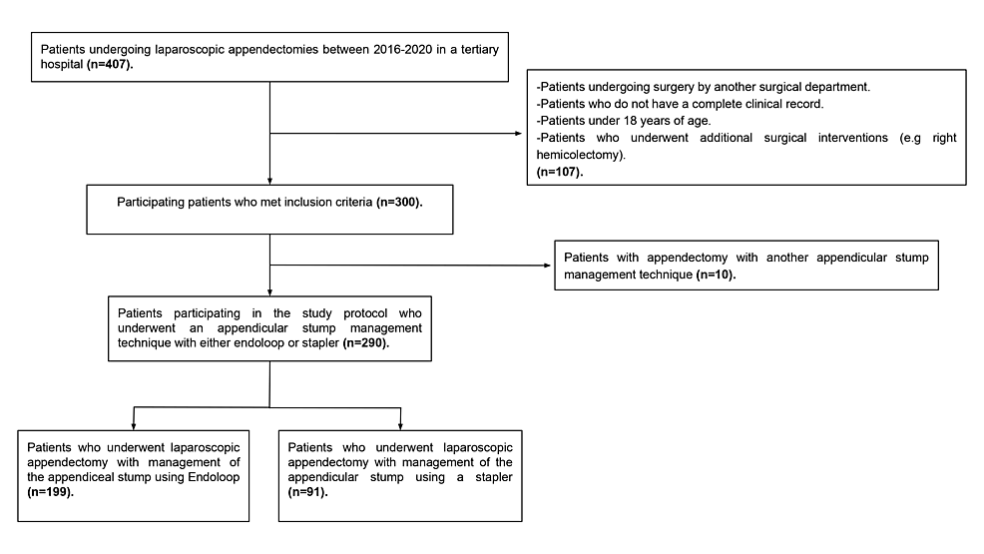
Flowchart showing patient selection

A retrospective cohort study in which data from patients between 18 and 83 years of age with a mean of 39.8 years (SD 1.3 +/-) undergoing laparoscopic appendectomy by the general surgery department of a tertiary care hospital in Mexico City, within the period between 2016 and 2020, were recorded. Patients undergoing surgery by another surgical department, patients who did not have a complete clinical record, patients under 18 years of age, patients with a different technique for the closure of the appendiceal stump different from the study variables (extracorporeal knot), and patients who underwent additional surgical interventions (e.g., right hemicolectomy) were excluded, thus obtaining a total of 290 patients (Figure 1). Data collection included demographic data, clinical history, tomographic and laboratory findings, type of appendicular base management technique (endoloop, endostapler, or other), and the severity of appendicitis at the time of hospital admission. The Alvarado score and American College of Surgeons-National Quality Improvement Program Surgical Risk Calculator (NSQIP) were calculated based on the patient's clinical record. Postoperative outcomes include complications at 30 days, readmission at 30 days, and an in-hospital stay. The latest available version of Windows Excel (Microsoft Corporation, Redmond, WA, USA) was used for data collection. For categorical variables, Fisher's exact test and Pearson's Chi-square test were used. Student's t-test was used for numerical variables with a normal distribution, and Mann-Whitney's U test was used for numerical variables without a normal distribution. The Shapiro-Wilk test was used to confirm a normal distribution of numerical values. P-values <0.05 were considered statistically significant. A binary logistic regression was carried out using the forward step method (LR), based on partial maximum likelihood estimates, to identify the risk factors, and only the variables that gave significance in the univariate analysis were included. All statistical analyses were performed using SPSS Statistics (IBM Corp., IBM SPSS Statistics for Windows, Armonk, NY: IBM Corp.).

## Results

Patient demographics at the time of hospital admission are shown in Table 1. The mean age was similar in both groups, being 35 and 40 years in endoloop and endostapler, respectively (p=0.096, 95% CI). The distribution between males and females in both groups was similar and did not represent a significant variable (p=0.128, 95% CI). Clinical history, the presence of fever, and BMI did not have statistically significant differences (p≥0.05). Clinical findings on hospital admission, scales (Alvarado and NSQIP), degree of appendicitis (severity level), and tomographic findings are shown in Table 2. The only statistically significant variable was the presence of a pre-surgical abscess in tomographic findings, which was 1.58 times higher in the endostapler group (p=0.034, 95% CI, SD 0.937-2.664) than in the endoloop group. Post-surgical results at 30 days are presented in Table 3. The overall mean of in-hospital stay was the same in both groups, with an average of two days (p=0.244, 95% CI). There is a higher number of patients with hospital readmission at 30 days in the endostapler group compared to the endoloop group, with 5 (5.5%) and 3 (1.5%) patients, respectively. However, no statistically significant difference was found (p=0.114, 95% CI). It was found that the use of endostapler devices presented 2.7 times more risk of postoperative abscess compared to the use of an endoloop (p=0.000, 95% CI, SD 1.289-5.764). Similarly, it was found that the endostapler group had 1.87 times more risk of postoperative sepsis (p=0.011; 95% CI, SD 0.993-3.552). The rest of the post-surgical complications did not provide statistically significant data. The results of binary logistic regression for postoperative abscess factors are reported in Table 4. Preoperative abscess increased the probability of postoperative abscess by 15.9 times (95% CI, SD 4.8-52.3), independent of the closure technique used. The endoloop method reduced the risk of postoperative abscess by 16.5% (95% CI, SD 0.054-0.505), while the endostapler method increased the risk of postoperative abscess at the right iliac fossa by six times (95% CI, SD 1.98-18.60). The results of binary logistic regression for postsurgical sepsis factors are reported in Table 5. The presence of a preoperative abscess increased the risk of postoperative sepsis by 7.20 times (95% CI, SD 2.09-24.74), while the endoloop method protects by 30% against the development of post-surgical sepsis (95% CI, SD 0.104-0.900). The endostapler method increased the risk of postoperative sepsis by 3.2 times (95% CI, SD 1.11-9.62).

**Table 1:** Demographic variables at the time of hospital admission The statistically significant p-value <0.05 is indicated in bold. For categorical variables, Fisher's exact test and Pearson's Chi-square test were used. For numerical variables with normal distribution, Student's t-test was used. For numerical variables without normal distribution, Mann-Whitney's U test was used.

Variable		Endoloop (n=199)	Stapler (n=91)	p-value
Age (years) (mean, range)		35.0 (26.0-50.0)	40.0 (31.0-54.0)	0.096
Sex (N (%))	M = male	M 110 (55.3%)	M 41 (45.1%)	0.128
	F = female	F 89 (44.7%)	F 50 (54.9%)	
Positive smoker in the year of surgery (N (%))		72 (36.2%)	27 (29.7%)	0.289
Type 2 diabetes (N (%))		6 (3.0%)	7 (7.7%)	0.121
High blood pressure that requires medication (N (%))		18 (9.0%)	12 (13.2%)	0.302
Pre-surgical sepsis (N (%))		35 (17.6%)	22 (24.2%)	0.205
Chronic steroid use (N (%))		4 (2.0%)	2 (2.2%)	1.000
Acute renal failure (N (%))		3 (1.5%)	1 (1.1%)	1.000
Systemic inflammatory response syndrome (N (%))		61 (30.7%)	22 (24.2%)	0.327
Presence of fever (N (%))		41 (29.6)%	19 (20.9%)	1.000
Body mass index (kg/m2) (mean, range)		24.97 (22.65-28.36)	25.07 (22.60-28.72)	1.000

**Table 2:** Clinical findings on hospital admission The statistically significant p-value <0.05 is indicated in bold. For categorical variables, Fisher's exact test and Pearson's Chi-square test were used. For numerical variables with normal distribution, Student's t-test was used. For numerical variables without normal distribution, Mann-Whitney's U test was used. NSQIP: The Alvarado score and American College of Surgeons-National Quality Improvement Program Surgical Risk Calculator

Variable		Endoloop (n=199)	Stapler (n=91)	p-value
Alvarado scale (mean, range)		7.0 (5.0-8.0)	7.0 (5.0-9.0)	0.364
NSQIP preoperative evaluation (mean, range)		3.80 (3.50-5.30)	3.90 (3.50-5.50)	0.739
Appendicitis phases (N (%))	I	I 76 (38.2%)	I 32 (35.2%)	0.158
	II	II 122 (61.3%)	II 56 (61.5%)	
	III	III 1 (0.5%)	III 3 (3.3%)	
	IV	IV 0 (0%)	IV 0 (0%)	
Leukocytes (x109/L) (mean +/-SD)		12,772 (+/-3.77)	13,024 (+/- 4.57)	0.646
Striation of fat at tomographic findings (N (%))		176 (88.4%)	82 (90.1%)	0.840
Presence of abscess at tomographic findings (N (%))		8 (4.0%)	10 (11.0%)	0.034
Presence of open air at tomographic findings (N (%))		10 (5.0%)	8 (8.8%)	0.293
Presence of free fluid at tomographic findings (N (%))		58 (29.1%)	27 (29.7%)	1.000
Appendicular diameter by tomography (mm) (mean, range)		11.4 (9.85-14.0)	12.50 (11.0-15.0)	0.089

**Table 3:** Post-surgical results at 30 days The statistically significant p-value < 0.05 is indicated in bold. For categorical variables, Fisher's exact test and Pearson's Chi-square test were used. For numerical variables with normal distribution, Student's t-test was used. For numerical variables without normal distribution, Mann-Whitney's U test was used. NC: not calculable

Variable	Endoloop (n=199)	Stapler (n=91)	p-value
Length of in-hospital stay (days) (mean, range)	2 (2-3)	2 (1.5-3)	0.244
Re-entry = 30 days (N (%))	3 (1.5%)	5 (5.5%)	0.114
Presence of post-surgical abscess (N (%))	5 (2.5%)	14 (15.4%)	0.000
Presence of residual stump leakage (N (%))	0 (0%)	0 (0%)	NC
Presence of post-surgical sepsis (N (%))	6 (3.0%)	10 (11%)	0.011
Presence of post-surgical hemorrhage/hematoma (N (%))	2 (1.0%)	0 (0%)	1.000
Other post-surgical complications (N (%))	5 (2.5%)	6 (6.6%)	0.106

**Table 4:** Binary logistic regression for postsurgical abscess factors The statistically significant p-value <0.05 is indicated in bold. Binary logistic regression was carried out using the forward step method (LR). RR: relative risk

Variable	RR	Confidence interval (95%)	p-value
Pre-surgical abscess	15.923	4.8-52.3	0.000
Endoloop	0.165	0.054-0.505	0.002
Endostapler	6.060	1.97-18.602	0.002

**Table 5:** Binary logistic regression for postsurgical sepsis factors The statistically significant p-value <0.05 is indicated in bold. Binary logistic regression was carried out using the forward step method (LR). RR: relative risk

Variable	RR	Confidence interval (95%)	p-value
Post-surgical sepsis	7.202	2.09-24.74	0.002
Endoloop	0.306	0.104-0.900	0.032
Endostapler	3.378	1.11-9.62	0.032

## Discussion

Currently, several methods are available for closing the appendiceal stump, which is the critical point when performing both laparoscopic and open appendectomy. Regarding these methods, endostaplers seem to be among the most widely used closing methods; however, our study shows them to be associated with high rates of postoperative complications, which is consistent with other studies, and the associated costs of these complications limit their use in all but the most severe cases of appendicitis [5]. Detailed results have also been obtained by comparing the use of endoloop and endostapler devices. In our study, a higher number of patients with hospital readmission at 30 days was obtained in the endostapler group compared to the endoloop group, with five (5.5%) and three (1.5%) patients, respectively. In their study involving 6,486 patients diagnosed with appendicitis, Beldi et al. noted a significant trend: among the 2,565 patients who underwent laparoscopic appendectomy with endoloops for appendicular stump closure, there was a notable occurrence of hospital readmission. Moreover, they identified intra-abdominal complications (intra-abdominal abscesses) as the second most common cause of readmission within this patient group [8].

On the other hand, Sahm et al. observed a lower incidence of intra-abdominal abscesses with the use of endoloops compared to the use of endostapler in their retrospective analysis of 1,790 patients [9]. There is currently no consensus on the number of endoloops recommended for appendiceal stump closure. In a study of 208 patients, in which 109 patients had their stump closed with one endoloop and 99 patients had their stump closed with two endoloops, intra-abdominal abscess formation subsequently occurred in group 1 in three patients (4.6%), while in group 2 it occurred in four patients (5.1%), with no significant differences between the two groups [10].

It was also found that the endostapler group in our study had a 1.87 times higher risk of post-surgical sepsis. It was also found that the use of endoloop reduced the risk of post-surgical abscess by 16.5% (95% CI, SD 0.054-0.505), while the use of endostapler devices increased the risk by six times (95% CI, SD 1.98-18.60). Gomes et al. reported that in their cohort comparing the use of metal endoclip with the use of endoloop, the frequency rate of intra-abdominal abscess formation was 5.4% [6], while Makaram et al. reported that the use of endostapler devices had a 13.6% rate for intra-abdominal abscess formation in the postoperative period [5]. In a study conducted by Safavi et al. involving 242 patients who underwent laparoscopic appendectomy, 24 patients had their appendicular stump closed with an endostapler device, while 218 patients underwent closure with an endoloop. The study revealed a notably higher incidence of intra-abdominal abscess formation in the group that underwent endostapler closure compared to those with endoloop closure [11].

Despite the data presented in the literature, Beldi et al. reported that there was a greater occurrence of intra-abdominal abscess in postoperative patients undergoing laparoscopic appendectomy with the closure of the appendiceal stump using endoloop than in the use of endostapler devices group, where they recommended the use of endostapler devices [8]. However, within their results of intraoperative and postoperative complications, the group where an endostapler device was used had a higher number of complications, and only the endoloop group had a statistically significant result (p=0.002), which was the rate of hospital readmission. Similarly, Kazemier et al. concluded that the use of endoloops prolongs the surgical duration by nine minutes, potentially leading to unexplored complications in this study. They also describe that the use of endoloops requires surgical expertise to establish the knot and adjust the tension; the use of endoloops can cause tissue slippage and cuts, possibly leading to stump leakage as a result of local necrosis; whereas the use of an endostapler device is safer because there is a clear reduction of wound infection and postoperative ileus [12,13].

The use of an endostapler is therefore recommended in limited cases, such as severe inflammation or when the appendicular base is more than 10 mm in diameter. The endoloop, on the other hand, is preferred in an appendicular base smaller than 10 mm and when the inflammation is not severe [14]. Escolino et al. reported in their study on the treatment of appendiceal stump closure in complicated appendicitis that group 1 using endoloops included 374 patients (52.8%) and group 2 using endostapler devices included 334 patients (42.2%). The group using endoloops was significantly associated with a higher incidence of intra-abdominal abscesses (OR 1.36; 95% CI: 0.84-2.18) and postoperative ileus (OR 3.6; 95% CI: 0.76-17.11) compared to endostapler devices [15].

The current literature suggests that the use of endoloops provides an effective and easy-to-perform closure technique with a low risk of intraoperative closure complications [16]. It has been hypothesized that a higher risk of abscess formation exists secondary to exposure to contaminated mucosa following the use of endoloops. However, this is not consistent with our study, where most complications were prevented by the preference of endoloops over endostapler devices.

Some limitations inherent in our study include material availability, where we had more options and flexibility in utilizing endoloops compared to mechanical staplers. Cost is also a significant consideration, as the utilization of endoloops proves to be more economical than mechanical staplers. Furthermore, surgeon preference and skill are notable factors, given that both methods (endoloop and mechanical staplers) entail a learning curve for the surgeon.

## Conclusions

Based on the results presented in this study, it was determined that the group where endoloop was used reduced the risk of postoperative abscess by 16.5%, while with the use of an endostapler device, the risk of postoperative abscess increased six times. These results coincide with the majority of the literature consulted. Furthermore, it was determined that the use of endoloop protects against the development of postoperative sepsis by 30%, while the use of endostapler devices increases the risk of postoperative sepsis by 3.2 times. However, it is proposed to individualize the use of endoloop and endostapler since the use of an endostapler is recommended in limited scenarios. Further studies of this nature, with a larger sample size and a broader range of variables, are necessary to confirm these results with greater accuracy.
